# Exercise-Linked Irisin Prevents Mortality and Enhances Cognition in a Mice Model of Cerebral Ischemia by Regulating Klotho Expression

**DOI:** 10.1155/2021/1697070

**Published:** 2021-07-08

**Authors:** Zhao Jin, Zongze Zhang, Jianjuan Ke, Yanlin Wang, Huisheng Wu

**Affiliations:** ^1^Department of Anesthesiology, Zhongnan Hospital of Wuhan University, Wuhan City, Hubei Province 430071, China; ^2^Department of Anesthesiology, The First Affiliated Hospital of Anhui Medical University, Hefei City, Anhui Province 230022, China

## Abstract

Irisin, which can be released in the hippocampus after physical exercise, is demonstrated to have beneficial effects on neurovascular diseases. This study investigated the impact of exercise linked-irisin on mortality and cognition in a mice model of cerebral ischemia and further explored its underlying mechanism. The cerebrospinal concentrations of irisin and klotho from ischemic stroke patients were measured with an enzyme-linked immunosorbent assay (ELISA). The cognitive function of mice was evaluated by a series of behavioural experiments. The expressions of klotho, MnSOD, and FOXO3a in the hippocampus of mice were detected by Western blot. Superoxide production in the brain tissue of mice was evaluated with the dihydroethidium (DHE) dying. The results demonstrated that stroke patients showed a positive correlation between their CSF irisin concentration and klotho concentration. In addition, when mice subjected to cerebral ischemia, their cognitive function was impaired, the protein expressions of klotho, MnSOD, and FOXO3a downregulated, and the production of reactive oxygen species (ROS) increased compared with the sham group. After pretreatment with exogenous irisin, improved cognitive impairment, upregulated protein expressions of klotho, MnSOD, and FOXO3a, and reduced ROS generation were observed in mice with MCAO. However, the neuroprotective effects of irisin compromised with the evidence of severe cognitive impairment, decreased protein expressions of MnSOD and FOXO3a, and increased ROS production in klotho knockout mice. Thus, our results indicated that exercise-linked irisin could prevent mortality and improve cognitive impairment after cerebral ischemia by regulating klotho expression.

## 1. Introduction

The incidence of ischemic stroke, which is the second cause of mortality and dementia in older individuals, is increasing annually worldwide [1, 2]. More than 50% of stroke patients will have cognitive impairment in clinic, and about 10% of them will have dementia, which seriously affects their prognosis and quality of life [3, 4]. Therefore, the need for effective therapeutic interventions to treat ischemic stroke remains urgent. More and more efforts are focused on exploring strategies to counteract mechanisms causing cognitive impairment and neuronal damage.

It is well-established that physical exercise can exert neuroprotective effects on some neurological disorders, with the underlying mechanism remained to be elucidated [5, 6]. Irisin, which can be released into circulation after physical exercise, is identified as a myokine [7]. It is cleaved from fibronectin type III domain-containing protein 5 (FNDC5) by proteolytic enzyme. Recent research has demonstrated that irisin can stimulate adipocyte browning and work as a neuroprotectant for various neurological diseases [8]. In addition, the cognitive function could be improved by exercising and by promoting brain-derived neurotrophic factor (BDNF) in the hippocampus via the PGC-1*α*/FNDC5 pathway [9].

What is more, FNDC5/irisin could rescue memory defects and synaptic plasticity in Alzheimer's models [6]. Our previous studies also demonstrated that exogenous irisin could alleviate neuronal apoptosis and prevent cerebral ischemia/reperfusion injury [10]. However, the underlying mechanism remained to be investigated.

Klotho discovered by Kuro-o is identified initially as an ageing-regulator gene [11]. It is mainly expressed in the kidneys and choroid plexus [12–14]. Recent studies have revealed that the klotho protein plays a critical role in delaying ageing and enhancing cognition [15–18]. Klotho mutant mice exhibit a shortened lifespan and impaired synaptic integrity and awareness, whereas klotho overexpression extends the lifespan and improves synaptic integrity and cognitive impairment in mice [19]. Besides, some clinical researches have demonstrated that the content of klotho in the cerebrospinal fluid (CSF) of the elderly is lower than that of young people. Its mutation is closely related to cognitive dysfunction in the elderly [20]. Meanwhile, klotho protein concentration in the CSF of patients with Alzheimer's disease was also significantly reduced [21]. We found a positive correlation between the CSF irisin level and the CSF klotho level of stroke patients in the preexperiment. Thus, we explored whether exogenous irisin exerted neuroprotective effects on cerebral ischemia by regulating klotho expression.

Our present study demonstrated that the neuroprotective effects of physical exercise and exogenous irisin on cerebral ischemia were the same, and irisin could prevent mortality and improve cognitive dysfunction after cerebral ischemia through upregulating the expression of klotho.

## 2. Materials and Methods

### 2.1. Clinical Study of Stroke Patients

Fourteen patients with ischemic stroke were recruited from the neurology department at the Zhongnan Hospital of Wuhan University. Samples of cerebrospinal fluid (CSF) from ischemic stroke patients during acute illness and after recovery were collected. Besides, twenty control CSF samples were collected from vertigo patients in the neurology department at the Zhongnan Hospital of Wuhan University, excluding those with stroke, neurodegenerative disorders, epilepsy, or other neurological diseases. The stroke diagnosis was defined according to the international standard. The concentrations of irisin and klotho in CSF were measured by ELISA. The cognition of stroke patients was evaluated by the Montreal Cognitive Assessment (MoCA) Test immediately after their samples of CSF were collected. All procedures were performed according to the protocol approved by the Ethics Committee of Zhongnan Hospital of Wuhan University, and informed consent was obtained from all participants and their legal representatives (registration number: ChiCTR2000038569, ethics batch number: 2018006).

### 2.2. Animals

Heterozygous klotho mutant mice (C3H) with C57BL/6J background were hybridized to obtain wild-type and klotho mutant mice. Then, eighty-four mice weighing 20–25 g were placed in the same environment for more than three days where the temperature was 20-25°C, and the light/dark cycle was 12 h. They were allowed to take in food and water freely. Before the behavioural experiment, the mice adapted to the environment for a week. The experimental procedures were approved by the Animal Care and Use Ethics Committee of Wuhan University and conducted in line with the National Institutes of Health Guide for the Care and Use of Laboratory Animals.

### 2.3. Aerobic Exercise in Mice

Mice swam in a plastic bucket (60 cm depth × 50 cm diameter), and the water temperature was maintained at 23-25°C. The adaptive training lasted for seven days to reduce water-induced stress. Then, for the first three days, mice were adapted to swimming for 20 min, and the duration extended for 10 min day by day until the daily exercise time reached 60 min. After training, swimming was performed from 3 pm to 5 pm, and the duration was 60 min, five days per week for six weeks or four weeks according to different experimental requirements.

### 2.4. MCAO Mouse Model

Induction of middle cerebral artery occlusion (MCAO) was performed as described previously [22]. Briefly, mice were anaesthetized with 80 mg/kg pentobarbital intraperitoneally and then fixed in the supine position. The right common carotid artery, internal carotid artery, and external carotid artery were separated after median skin incision. The branches of the external carotid artery were cut off by electrocoagulation. A 4-0 nylon thread with a round head was inserted into the internal carotid artery from the common carotid artery and advanced until there is a slight sense of resistance. After 2 h of cerebral ischemia, the nylon thread was withdrawn to the stump of the external carotid artery to form reperfusion. Mice in the sham group underwent the same surgery, except thread insertion. All surgical procedures were conducted under sterile conditions.

### 2.5. Experimental Protocol

The whole animal experiment could be divided into three parts. The first part was designed to determine whether irisin mediated the neuroprotective effects of physical exercise on cognition in a mice model of MCAO. Mice were randomly divided into six groups:(1) sham group (S), mice subjected to the sham operation; (2) MCAO group (MCAO), mice underwent occlusion of the right middle cerebral artery; (3) irisin-treated group (Ir), mice received an intravenous injection of 10 *μ*g/kg irisin 30 min before MCAO; (4) swimming group (SW), mice underwent four weeks of regular swimming before MCAO; (5) SW+control IgG group, mice underwent four weeks of regular swimming and then treated with nonimmune control IgG; (6) SW+irisin NA group, mice underwent four weeks of regular swimming and then treated with an irisin neutralizing antibody. The control IgG or irisin neutralizing antibody was injected intravenously 1 h before MCAO. Then, mice were trained for the following behavioural tests.

The second part was conducted to explore the mechanism of the neuroprotective effects of exogenous irisin. Mice were randomly divided into three groups: sham group (S), MCAO group (MCAO), and irisin-treated group (Ir). The interventions for these three groups were consistent with those in the first part and mice were sacrificed at 24 h after MCAO. The hippocampus was dissected to detect the contents of reactive oxygen species (ROS) and the expression of klotho.

The last part of the experiment was designed to further determine the relationship between the neuroprotection of irisin and klotho expression. Mice were randomly divided into two groups: the wild-type group (WT) and the klotho knockout group (KO). Mice in both groups were injected 10 *μ*g/kg irisin intravenously, and 30 min later underwent the right middle cerebral artery occlusion. Behavioural tests were conducted at 24 h after MCAO, then mice were sacrificed, and the hippocampal tissues were collected to detect the contents of ROS.

### 2.6. Behavior Testing

#### 2.6.1. Morris Water Maze

Morris water maze (MWM) test was mainly used to test spatial learning and memory ability [19]. The water maze pool was 100 cm in diameter, 50 cm high, and 30 cm in depth. Then, white, opaque water at a temperature of 23 ± 2°C was put into the pool with plenty of spatial clues around it. A platform of 12 cm in diameter was placed underwater, making it invisible. At first, mice underwent hidden platform training conducted four times a day for five days. Each time mice were put into water from different quadrants. If mice could not find the platform within the 60 s, the experimenter would guide the mice to the platform and rest on it for 10 s before the subsequent trial, and the latency was recorded as 60 s. An automated video recording system recorded the escape latency and the swimming distance. After the hidden platform training, the platform was removed, and the probe trial was followed. In the probe trial, the retention time of the mice spent in the target quadrant was recorded.

#### 2.6.2. Open Field Test

An open field test was used to evaluate the autonomous behaviour, the exploratory behaviour, and tension of mice in a new and different environment [19]. Mice were placed in the centre of the bottom of the box (45 × 45 × 30 cm^3^) and observed for 5 min. The total distance travelled by the mice (horizontal activities) and rearings (vertical activities) were recorded by an automated camera-based computer tracking system. The inner wall and the bottom surface of the apparatus were cleaned to avoid the information left by the last animal (such as the mice's urine and smell) from affecting the following test result.

#### 2.6.3. Novel Object Recognition Task

The novel object recognition (NOR) task provided a valuable method to evaluate recognition memory in mice [19]. In this test, mice were placed in an open field arena with two objects with the same volume and height but different appearance and shape. At first, mice were exposed to an empty arena for 24 h to adapt to the environment. Then, mice were allowed to explore the familiar arena with two identical objects placed at the same distance for 5 min. The next day, the mice were placed back into the same arena in the presence of two objects, with one of them replaced for a novel object for another 5 min. The time that the mice spent exploring the novel object was recorded.

#### 2.6.4. Fear Conditioning Test

The fear conditioning test (FCT) was used to assess the capacity of learning and memory in mice [23]. It consisted of a training phase and a test phase. In the training phase, mice were placed into the training chamber for 3 min. Then, they received five repeated pairings of the conditional stimulus (CS, often a tone, 75 dB, 2 kHz, 20 sec) and the unconditional stimulus (US,0.5 mA, 2 sec). The interval between the tone and the foot shock was 18 seconds, and the gap between the pairs of conditional-unconditional stimuli was 45-60 seconds. In the test phase, mice were placed into another chamber for 3 min and subsequently given three-tone presentations without any shocks for the tone-dependent test. On the second day, mice were placed back into the training chamber for 5 min without any CS and US for the context-dependent test. The times of the freezing response were measured during the whole experiment.

### 2.7. Immunohistochemistry

The paraffin sections (5 *μ*m thick) of mouse brain tissue were roasted overnight at 37°C. Then, the sections were deparaffinized and incubated with 3% hydrogen peroxide for 15 min. After the thermal repair of the tissue antigens by microwave, the slices were cooled for 30 min at room temperature and incubated with mouse antinuclear monoclonal antibody and rabbit anti-klotho and anti-irisin polyclonal antibodies. Then, the protein expressions of klotho and irisin were observed after biotin-labelled secondary antibody incubation. Five nonoverlapping visual fields around the infarct of each slice were observed under a light microscope (×400) by a blind pathologist. The integrated optical density (IOD) was measured by an Image-Pro Plus analysis system. The protein expression intensity was positively correlated with IOD [24].

### 2.8. Western Blotting

The proteins from the damaged brain tissues were extracted after MCAO following the manufacturer's instructions [19]. Then, the proteins were separated by SDS-polyacrylamide gel electrophoresis onto the nitrocellulose membrane and buffered and blocked with skimmed milk powder at 4°C overnight. Next, the membranes were incubated with the primary antibodies against klotho, MnSOD, FOXO3a, or phosphorylated FOXO3a. Finally, after being washed with PBS twice, the membranes were incubated with the secondary antibodies at room temperature for 2 h. The density results were analyzed by Image-J software.

### 2.9. ELISA

The enzyme-linked immunosorbent assay (ELISA) was used to determine the levels of irisin and klotho protein in the CSF of controls and stroke patients at acute and recovery stages. The procedure was carried out according to the manufacturer's instructions using the ELISA kit (Boster Biological Technology Co. Ltd., Wuhan).

### 2.10. DHE Staining

Superoxide production in the mouse brain tissue was evaluated with the dihydroethidium (DHE) dying [25]. Frozen sections of the damaged brain tissue isolated from mice were incubated with DHE at 37°C for 40 min and fixed with paraformaldehyde for 10 min. Then, DAPI staining solution was used to stain the nucleus for 10 min. Finally, the images were observed using a fluorescence microscope at an excitation wavelength of 490 nm and an emission wavelength of 590 nm. The blue-stained part was the nucleus, and the green fluorescence reflected the ROS content. The exposure time used for image acquisition of all sections was 30 ms. The intensity of DHE fluorescence was quantified by an analysis system.

### 2.11. Statistical Analysis

All data were presented as mean ± S.E.M. Repeated measure analysis of variance (ANOVA) was used for comparison at different time points within groups, and ANOVA with Tukey's test was used for multiple comparisons among groups. The significance of correlations was evaluated by determining Spearman's rank correlation coefficients. The difference of estimate of cumulative survival (Kaplan–Meier) among groups was assessed with the log-rank test. A value of *P* < 0.05 was indicated significant. Statistical analysis was carried out using SPSS 20.0 (SPSS Inc., San Rafael, CA, USA) and GraphPad Prism 5.0 (San Diego, CA, USA).

## 3. Results

### 3.1. Stroke Patients Show a Positive Correlation between Irisin and Klotho Concentration in Their CSF

**Figure 1:**
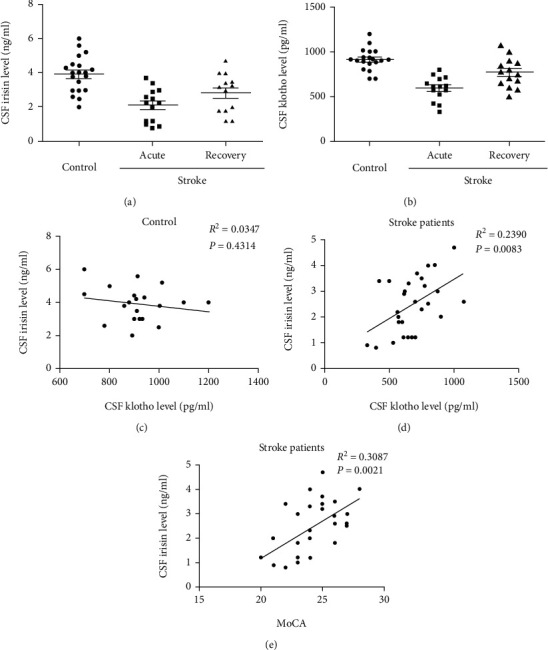
Stroke patients showed a positive correlation between irisin and klotho concentration in their CSF. The CSF irisin concentration (a) and klotho concentration (b) were detected by ELISA. There was no correlation between the CSF irisin concentration and the CSF klotho concentration for controls (c). At the same time, there was a positive correlation between the CSF irisin concentration and the CSF klotho concentration for stroke patients (d). Stroke patients also showed a positive correlation between the CSF irisin concentration and their cognition (e). Data are expressed as mean ± S.E.M (*n* = 20 controls, 14 stroke patients).

We conducted a pilot human study to establish the relationship between the CSF irisin concentration and the CSF klotho concentration in ischemic stroke patients. A total of twenty controls and fourteen stroke patients were enrolled in the study. The CSF samples of controls and stroke patients were collected. Using ELISA, we found that the CSF irisin concentration and the CSF klotho concentration derived from stroke patients were lower than those derived from controls (*P* < 0.05, Figures 1(a) and 1(b)). On average, the CSF of controls contained 3.92 ± 0.23 ng/ml irisin and 912.8 ± 26.7 pg/ml klotho, the CSF of stroke patients at acute stage contained 2.12 ± 0.25 ng/ml irisin and 590.5 ± 36.3 pg/ml klotho, and the CSF of patients after recovery from stroke contained 2.83 ± 0.29 ng/ml irisin and 766.4 ± 43.8 pg/ml klotho. In addition, using correlation analysis, we determined that stroke patients showed a positive correlation between irisin and klotho concentration in their CSF while controls did not show any correlation (Figures 1(c) and 1(d)).

At the same time, the cognition of stroke patients was evaluated using the Montreal Cognitive Assessment (MoCA) test. Then, the correlation analysis was used to analyze the relationship between the CSF irisin concentration and MoCA scores. The results showed a positive correlation between the CSF irisin concentration and their cognition (Figure 1(e)).

### 3.2. Swimming Increased the Content of Irisin in the Brain of Mice

**Figure 2:**
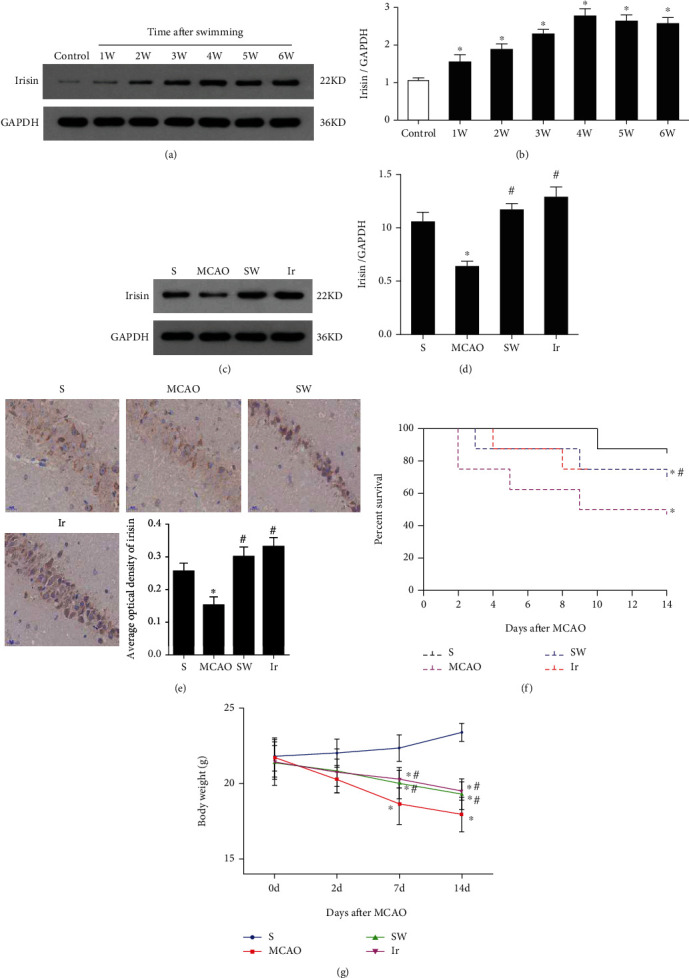
Swimming increased cerebral levels of irisin in mice. The expression of irisin in mice without MCAO after swimming for a different duration was detected by Western blot (a, b). The expression of irisin in the brain tissues of mice subjected to MCAO with or without swimming was detected by Western blot (c, d). Immunostaining showed that irisin was detected in the hippocampus of mice, and after swimming or exogenous irisin treatment, the expression of irisin in the hippocampus increased (e). Kaplan–Meier curves showed differences in survival rate among different groups (f), and the bodyweight of mice in the SW group and Ir group was higher than that in the MCAO group (g). Data are expressed as mean ± SEM, *n* = 6 per group, ^∗^*P* < 0.05 vs. S or Control, ^#^*P* < 0.05 vs. MCAO.

We initially observed the changes in the content of irisin in mice's brains after swimming at different times. Compared with controls, the content of irisin in the brain increased gradually for swum mice and reached a peak at the 4th week (*P* < 0.05, Figures 2(a) and 2(b)). It did not increase anymore in the following two weeks, which suggested that the content of irisin in mice's brains reached the peak at the fourth week of swimming, and there was no noticeable change after continuous exercise. Therefore, the swimming duration of mice in the following study was taken as 4 W.

Then, we tested whether a protocol of daily swimming could affect the brain levels of irisin in MCAO mice. Notably, compared with mice without exercise before MCAO, the expression of irisin in the brain increased when mice experienced 4 weeks of swimming before MCAO (*P* < 0.05, Figures 2(c) and 2(d)). All these results suggested that swimming could promote irisin production in the brain of mice with or without MCAO.

### 3.3. Swimming and Exogenous Irisin Improved Cognitive Impairment in Mice with MCAO

It has been demonstrated that exercise exerts a protective effect on cerebral ischemia-reperfusion injury and Alzheimer's disease [5], and irisin, as a critical peptide in the body, can be secreted by skeletal muscle and brain tissue after exercise [26]. This leads our study to observe whether physical exercise and exogenous irisin have the same neuroprotective effects on cerebral ischemia and whether irisin contributes to the neuroprotective effects of physical exercise.

To test this hypothesis, we first observed whether the expression of irisin in the brain changed after its exogenous administration. As shown in Figure 2(e), the expression of irisin could be detected in the hippocampus of mice by immunostaining. After swimming or being treated with exogenous irisin, the expression of irisin in the hippocampus significantly increased compared with the MCAO group (*P* < 0.05, Figure 2(e)). These results indicated that exogenous administration of irisin could promote it to enter the brain tissue.

To test whether swimming or irisin treatment could prevent mortality, we analyzed the survival rate of the MCAO mice that underwent swimming or were treated with exogenous irisin. The results revealed that both swimming and exogenous irisin improved survival in mice (*P* < 0.05, Figure 2(f)). In addition, animal weight is another objective indicator reflecting the survival of mice. The results showed that the bodyweight of mice in the swimming group and Ir group was higher than that in the MCAO group (*P* < 0.05, Figure 2(g)).

**Figure 3:**
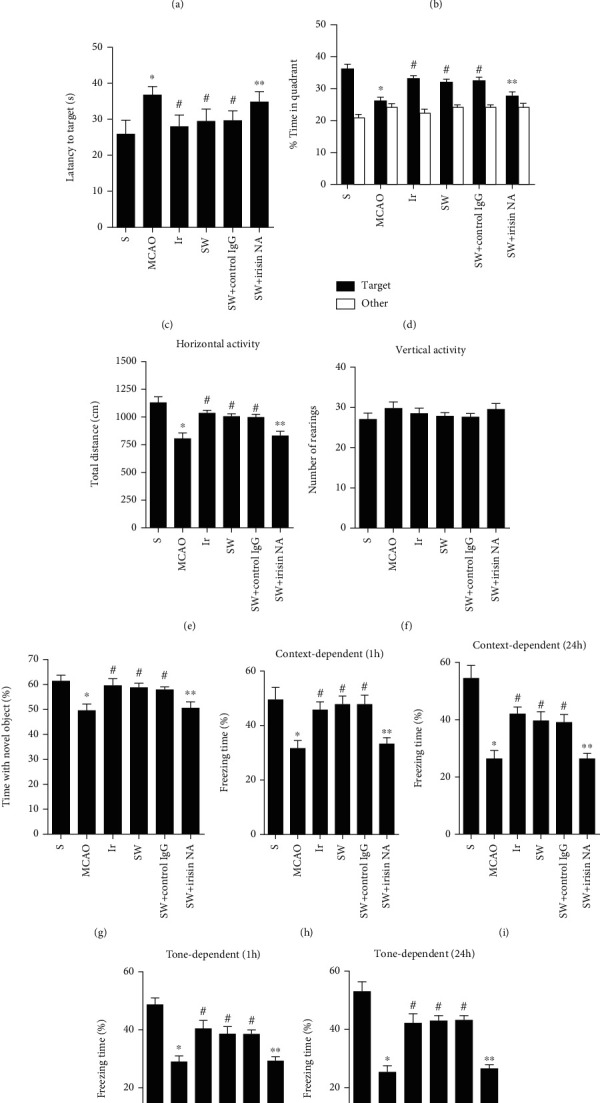
Swimming and exogenous irisin improved cognitive impairment in mice with MCAO. The experimental design was shown for the evaluation of the cognitive function of mice (a). Mice in different groups were tested in the Morris water maze test (b–d), in an open field test (e, f), a novel object recognition task (g), and a fear conditioning test (h–k). Data are expressed as mean ± S.E.M, *n* = 5 per group, ^∗^*P* < 0.05 vs. S,^#^*P* < 0.05 vs. MCAO, ^∗∗^*P* < 0.05 vs. SW+ control IgG.

To determine whether swimming and exogenous irisin could decrease deficits in spatial learning and memory in mice with MCAO, we tested mice in the Morris water maze test. In the hidden-platform test, the daily average of distance travelled to the platform, and the escape latency were recorded to assess spatial learning ability. Compared to the MCAO group, the distance that the mice travelled and the latency to targets were decreased after they were treated with irisin or swam for 4 w before MCAO. After the completion of hidden training, the platform was removed, and the spatial memory retention was evaluated in a probe trial. After swimming for 4 w or exogenous irisin treatment, mice spent more time in the target quadrant relative to other quadrants compared to the MCAO group. However, this improvement of exercised mice was partially blocked by the irisin neutralizing antibody (*P* < 0.05, Figures 3(b)–3(d)).

To determine whether swimming and exogenous irisin could affect locomotion and exploratory behaviours in mice with MCAO, we tested mice in an open field test. In the open field test, the total distance that mice travelled and the number of rearings were recorded. Compared to the MCAO group, mice in the Ir group and the SW group travelled a greater total distance. In contrast, this improvement of exercised mice was partially blocked by the irisin neutralizing antibody (*P* < 0.05, Figure 3(e)). There was no difference in vertical exploratory activities among these three groups.

A novel object recognition task was used to evaluate visual recognition memory which was related to the hippocampus. Compared to the MCAO group, mice in the Ir group and the SW group spend more time exploring the novel object. In contrast, this improvement of exercised mice was inhibited by the irisin neutralizing antibody (*P* < 0.05, Figure 3(g)).

A fear conditioning test was used to assess the associative fear memory. In a context-dependent test that was hippocampus-dependent, mice in the Ir group and the SW group showed more freezing responses at 1 h and 24 h after conditioning than the MCAO group (*P* < 0.05, Figures 3(h) and 3(i)). What is more, in a tone-dependent test that was hippocampus-independent, both swimming and exogenous irisin increased the freezing time at 1 h and 24 h after conditioning compared to the MCAO group (*P* < 0.05, Figures 3(j) and 3(k)). All these improvements of exercised mice were partially blocked by the irisin neutralizing antibody.

All these results indicated that physical exercise and exogenous irisin had the same neuroprotective effects on cognitive impairment after MCAO, and irisin might contribute to the neuroprotective effects of exercise. Thus, exogenous irisin was used as a replacement for exercise to explore the underlying mechanisms.

### 3.4. Irisin Upregulated the Expression of Klotho and Reduced Oxidative Stress in Mice with MCAO

**Figure 4:**
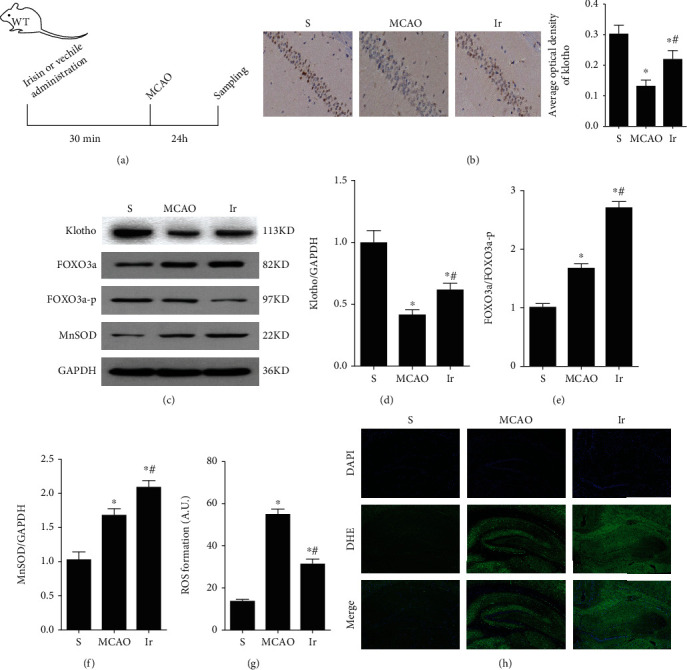
Irisin upregulated the expression of klotho and reduced oxidative stress in mice with MCAO. The experimental design was shown for the evaluation of the neuroprotective effects of irisin (a). Representative immunohistochemical images showed klotho expression in the hippocampus of mice in different groups (b). The expressions of klotho, FOXO3a, phosphorylated FOXO3a, and MnSOD were detected by Western blot (c–g). The extent of ROS production in the hippocampus of mice was determined by DHE staining (h). Data are expressed as mean ± S.E.M, *n* = 6 per group, ^∗^*P* < 0.05 vs. S, ^#^*P* < 0.05 vs. MCAO.

To further explore the mechanism of irisin improving cognitive dysfunction in mice with MCAO, we detected the expression of klotho and the indicators related to oxidative stress. Compared to the MCAO group, klotho protein expression was significantly upregulated when mice were pretreated with irisin (*P* < 0.05, Figures 4(b)–4(d)). In addition, the expression of forkhead transcription factor (FOXO3a) and manganese superoxide dismutase (MnSOD) increased, and the expression of phosphorylated FOXO3a decreased in the Ir group compared to those in the MCAO group (*P* < 0.05, Figures 4(e) and 4(f)). Using DHE staining, we found that mice subjected to MCAO showed enhanced fluorescence intensity, indicative of increased ROS generation. In contrast, in the Ir group, the fluorescence intensity decreased, indicating that exogenous irisin could significantly reduce ROS formation (*P* < 0.05, Figures 4(g) and 4(h)). All these results suggested that irisin could upregulate klotho expression and reduce oxidative stress in mice with MCAO.

### 3.5. Irisin Showed No Effect on Cognitive Function in Klotho Knockout Mice

**Figure 5:**
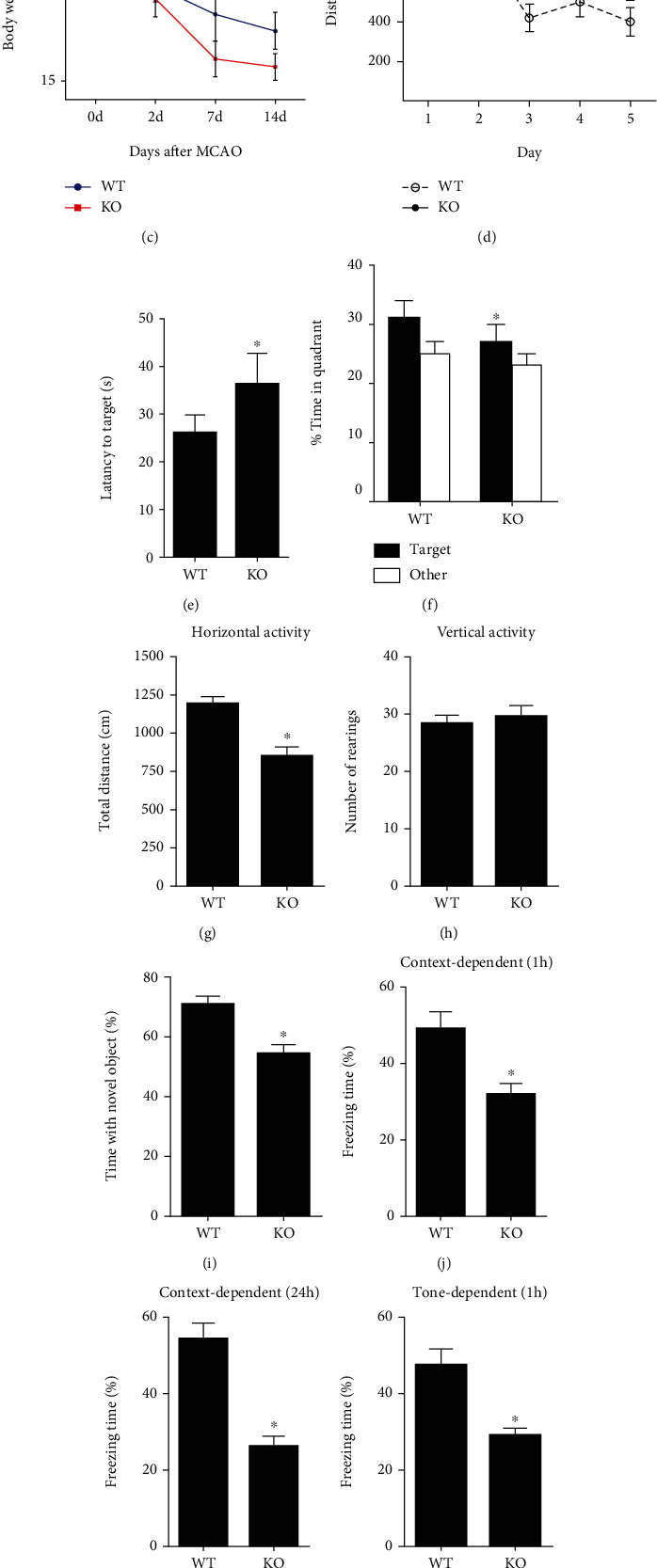
Irisin showed no neuroprotective effect on cognitive impairment in klotho^−/−^ mice. The experimental design was conducted for the evaluation of whether klotho mediated the neuroprotective effect of irisin (a). Kaplan–Meier curves showed differences in survival rate between WT mice and KO mice (b). The bodyweight of KO mice was lower than WT mice at 7 d and 14 d after MCAO (c). Mice in different groups were tested in the Morris water maze test (d–f). Mice in different groups were tested in an open field test (g, h), a novel object recognition task (i), and a fear conditioning test (j–m). Data are expressed as mean ± S.E.M, *n* = 6 per group, ^∗^*P* < 0.05 vs. WT.

**Figure 6:**
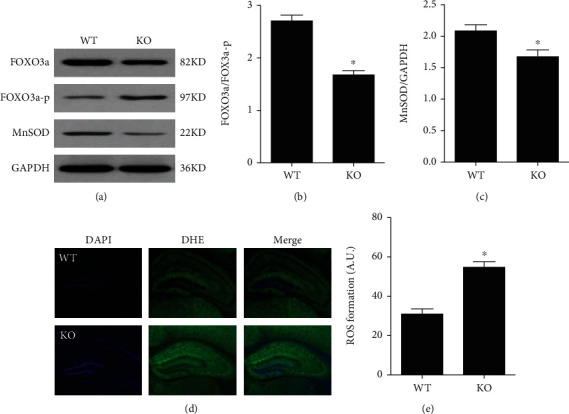
Irisin showed no neuroprotective effect on oxidative stress in klotho^−/−^ mice. The expressions of klotho, FOXO3a, phosphorylated FOXO3a, and MnSOD in the brain tissue of mice were detected by Western blot (a–c). The extent of ROS production in the hippocampus of mice was determined by DHE staining (d, e). Data are expressed as mean ± S.E.M, *n* = 6 per group, ^∗^*P* < 0.05 vs. WT.

To further explore the relationship between irisin and klotho for its improvement of cognitive function, we turned our attention to klotho knockout mice. Compared to KO mice, WT mice had lower mortality. The bodyweight of KO mice was lower than WT mice at 7 d and 14 d after MCAO (*P* < 0.05, Figure 5(c)). In the Morris water maze test, WT mice showed better spatial learning and memory than KO mice, which manifested that WT mice needed less time to find the hidden platform and spent more time in the target quadrant than KO mice (*P* < 0.05, Figures 5(d)–5(f)). In an open field test, WT mice travelled a greater total distance than KO mice (*P* < 0.05, Figure 5(g)). At the same time, no difference in the vertical exploratory activities was found between these two groups (Figure 5(h)). In a novel object recognition task, WT mice spend more time exploring the novel object compared to KO mice (*P* < 0.05, Figure 5(i)). In a fear conditioning test, WT mice showed more freezing responses at 1 h and 24 h after conditioning than KO mice for both context and tone dependent test (*P* < 0.05, Figures 5(j)–5(m)). In addition, the expression of FOXO3a and MnSOD decreased, whereas the expression of phosphorylated FOXO3a increased in the hippocampus of KO mice than in WT mice (*P* < 0.05, Figures 6(a)–6(c)). Using DHE staining, we found that KO mice showed enhanced fluorescence intensity, indicating increased ROS production than WT mice (*P* < 0.05, Figure 6(d)). All these results suggested that the neuroprotective effects of irisin on cerebral ischemia were compromised with the absence of the klotho gene.

## 4. Discussion

Data presented in the present study demonstrated that exercise-linked irisin prevented mortality and enhanced cognition after cerebral ischemia-reperfusion injury. In stroke patients, the CSF concentration of irisin was positively correlated with their cognition, and it was also positively correlated with the CSF concentration of klotho. In MCAO mice, both swimming and systemic administration of exogenous irisin prevented mortality and improved cognitive impairment. The latter could upregulate the expression of klotho and alleviate oxidative stress. Mice with klotho gene knockout displayed increased susceptibility to cognitive impairment relative to the WT littermates. The protective effect of irisin on cognitive impairment after cerebral ischemia was compromised in the klotho^−/−^ mice. Based on these data, we concluded that exercise-linked irisin exerted beneficial effects on mortality and cognitive dysfunction through mechanisms involving the upregulation of klotho expression.

Irisin, which can be released into circulation after aerobic exercise, is identified as a myokine [26, 27]. It is cleaved from fibronectin type III domain-containing protein 5 (FNDC5) by the proteolytic enzyme [28]. In recent years, it has been found that irisin can make white adipocyte browning and has a neuroprotective effect. It exerts a similar protective effect to aerobic exercise on cerebral ischemia [29, 30]. In this study, the higher the CSF irisin levels in stroke patients, the better their cognitive function. Therefore, we further carried out animal experiments and found that irisin could reduce the cognitive impairment after cerebral ischemia in MCAO mice and improve the survival rate. As one of the hormones increased after exercise, we also observed whether the neuroprotective effect of exercise was related to irisin. The results showed that the cognitive impairment of MCAO mice was improved after swimming. If the neutralizing antibody of irisin was added to the mice after swimming, the degree of cognitive impairment was aggravated compared with that of the simple swimming group. This result suggested that irisin may be involved in the neuroprotective effect of exercise. Thus, we further investigated the possible mechanisms of irisin in improving cognitive dysfunction after cerebral ischemia.

Some studies demonstrated that it might be due to the increased release of reactive oxygen species (ROS) after the recovery of blood perfusion in the ischemic area, leading to lipid peroxidation and inflammatory reaction in the brain and then damaging the functional regions related to cognition [31, 32]. The contents of lipids and polyunsaturated fatty acids are abundant in the brain. When oxygen consumption is increased and endogenous antioxidant activity is low, oxidative stress occurs quickly. Oxidative stress generally occurs in the excessive ROS generation and/or the deficiency of the antioxidant defence system, that is, the imbalance between the oxidation system and the antioxidant system [33]. After ischemic stroke, ischemia and hypoxia lead to ATP depletion and mitochondrial dysfunction, resulting in a large number of ROS production [34]. At the same time, protein synthesis is blocked, which leads to a decrease in antioxidant enzyme production and then the increase of ROS production. This will aggravate the oxidative stress damage, leading to oxidative and/or nitro damage of lipid, protein, and DNA in neurons [35]. In this study, we observed that ROS content in the brain tissue of MCAO mice increased with the appearance of cognitive dysfunction. After treatment with exogenous irisin, the generation of ROS was reduced. Therefore, we speculated that irisin could improve cognitive dysfunction caused by cerebral ischemia by alleviating oxidative stress.

At the same time, the expression of klotho was detected in brain tissue to explore whether it is also involved in the neuroprotective effect of irisin. In recent years, it has been found that klotho protein is closely related to the central nervous system. Its deletion or mutation will lead to cognitive and memory impairment similar to the ageing phenomenon. When the klotho gene was knocked out in mice, its lifespan reduced and synaptic integrity and cognition impaired, whereas overexpression of klotho might enhance synaptic plasticity and improve learning and memory at different life stages [36, 37]. In this study, we found that the CSF concentration of klotho in stroke patients decreased, positively correlated with the CSF concentration of irisin in stroke patients. Therefore, we further investigated whether klotho participated in the neuroprotective effect of irisin in vivo experiments and found that klotho expression decreased in MCAO mice and exogenous irisin could upregulate the expression of klotho in the brain tissue of MCAO mice. Besides, exogenous irisin could improve the cognitive impairment of wild-type mice, but it could not improve the cognitive impairment in the klotho^−/−^ mice. That is to say, the neuroprotective effect of irisin was compromised in the klotho^−/−^ mice. Thus, all the results indicated that klotho might contribute to the neuroprotective effect of irisin.

It has been demonstrated that klotho protein could regulate the insulin/insulin-like growth factor-1(IGF-1) signalling pathway. Forkhead box O (FOXO) transcription factors, such as FOXO1, FOXO3a, and FOXO4, are negatively regulated by the insulin/IGF-1 signalling pathway [38]. When the insulin/IGF-1 signalling pathway is activated, Akt could be phosphorylated and activated, leading to phosphorylation of FOXOs. Then, phosphorylated FOXOs are inactivated and excluded from the nucleus. Recent researches reported that klotho protein could inhibit the insulin/IGF-1 signalling pathway, which in turn reduced the phosphorylation of FOXOs [39]. The FOXOs in the nucleus then directly bind to mitochondrial manganese-superoxide dismutase (MnSOD), the promoter of antioxidant enzymes, and upregulate its expression, thereby leading to the attenuation of the ROS production [40]. In this study, we found that exogenous irisin could upregulate the expression of FOXO3a and MnSOD in mice with MCAO, which then conferred resistance to oxidative stress. At the same time, compared to WT mice, the expression of FOXO3a and MnSOD decreased in the klotho^−/−^ mice and ROS production increased although they were treated with irisin. Thus, it was indicated that irisin could alleviate oxidative stress by upregulating klotho expression and thereby improving cognitive dysfunction.

Admittedly, the current study still needs further perfection in the following aspects. First, our pilot study with stroke patients only included a limited number of CSF samples. This may be a stimulus for future studies to establish a multicentre study for a larger sample size. Second, in this study, we observed the effect of irisin on the cognitive function within 14 days after stroke but not the long-term change of cognitive function after stroke. This appeals to further studies investigating the long-term change of cognitive function after stroke, which can be affected by irisin. Third, our present study demonstrated that irisin could improve cognitive impairment by upregulating klotho expression, but the exact mechanism of how irisin regulated klotho was undiscovered. Further researches are needed to reveal the underlying mechanisms.

**Figure 7:**
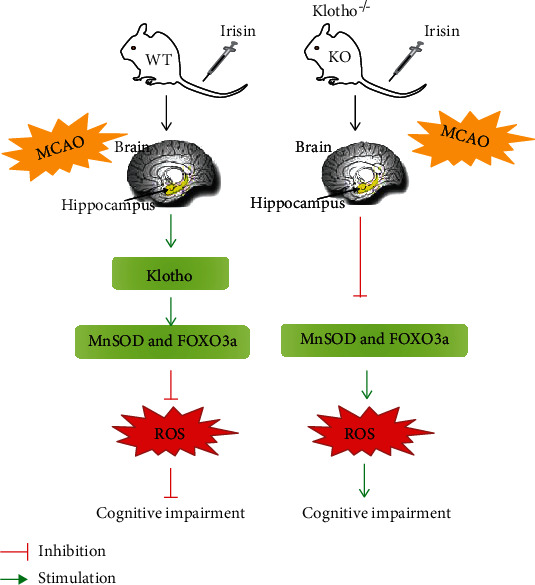
Proposed model for the roles of klotho protein regulated by irisin in the protection of cognitive impairment after MCAO.

In summary, the present study concluded that swimming could improve cognitive impairment in mice with MCAO by promoting the secretion of irisin in the brain tissue. Its effect might be similar to the treatment of exogenous irisin. At the same time, we also found that exogenous irisin could alleviate oxidative stress by upregulating klotho expression and thereby improving cognitive dysfunction after cerebral ischemic injury (Figure 7).

## Data Availability

The data used to support the findings of this study are available from the corresponding author upon request.
